# Knowledge-Aware Multispace Embedding Learning for Personalized Recommendation

**DOI:** 10.3390/s22062212

**Published:** 2022-03-12

**Authors:** Meng Jian, Chenlin Zhang, Xin Fu, Lifang Wu, Zhangquan Wang

**Affiliations:** 1 Faculty of Information Technology, Beijing University of Technology, Beijing 100124, China; mjian@bjut.edu.cn (M.J.); zhangchenlin@emails.bjut.edu.cn (C.Z.); 2 School of Water Conservancy and Environment, University of Jinan, Jinan 250022, China; stu_fux@ujn.edu.cn; 3 Inner Mongolia Aerospace Power Machinery Testing Institute, Huhhot 010076, China; zhangquanw@163.com

**Keywords:** collaborative filtering, user interest, knowledge graph, recommender system

## Abstract

Recommender systems help users filter items they may be interested in from massive multimedia content to alleviate information overload. Collaborative filtering-based models perform recommendation relying on users’ historical interactions, which meets great difficulty in modeling users’ interests with extremely sparse interactions. Fortunately, the rich semantics hidden in items may be promising in helping to describing users’ interests. In this work, we explore the semantic correlations between items on modeling users’ interests and propose knowledge-aware multispace embedding learning (KMEL) for personalized recommendation. KMEL attempts to model users’ interests across semantic structures to leverage valuable knowledge. High-order semantic collaborative signals are extracted in multiple independent semantic spaces and aggregated to describe users’ interests in each specific semantic. The semantic embeddings are adaptively integrated with a target-aware attention mechanism to learn cross-space multisemantic embeddings for users and items, which are fed to the subsequent pairwise interaction layer for personalized recommendation. Experiments on real-world datasets demonstrate the effectiveness of the proposed KMEL model.

## 1. Introduction

In the era of big data, people are surrounded by ubiquitous information. This has created the urgent need to filter the content that users require from the massive amount of available content. Personalized recommender systems help users find candidate content to meet users’ requirements, thereby alleviating the problem of information overload. Recommender systems have been widely applied in personalized music radio, e-commerce, multimedia platforms and other fields. As a core strategy, collaborative filtering refers to users’ historical interactions to predict users’ interests for personalized recommendation. However, it is difficult to capture their personalized preferences relying only on extremely sparse historical interactions. Personalized recommendation meets a significant challenge from sparsity, and cold start issues [1].

Fortunately, abundant semantic correlations exist among items that explain knowledge clues on users’ interests. For example, in the online movie application domain, the semantic relationship between both director and actor are crucial for capturing users’ interests. It is intuitive to involve semantic relations upon user–item interactions to alleviate the performance limitations from data sparsity and cold start issues. Some current studies [2,3,4,5] embed semantic associations in interest mining, which performs effectively for capturing users’ interests. Their success inspires us to think about the role of rich semantic correlations on recommendation tasks. As a hot topic, semantic correlation extraction has been actively studied in the field of knowledge graphs [6,7]. A knowledge graph, as an auxiliary structure, provides additional semantic correlations among items. Knowledge graphs also play a role to assist matching pairwise user–items by naturally integrating semantic correlation between items [8,9,10,11] strive to model users’ interests with high-order user–item semantic associations on the knowledge graph. Since knowledge graphs include huge-scale entities and diverse semantic relations, high-order mining may get lost in semantics for personalized recommendation; however, the semantic clue associated with the target user–item pair is worth noting.

We argue that the semantics in knowledge graphs make more sense than the large-scale entities on users’ interests. In this work, we investigate semantic correlations among items in view of multispace learning and propose knowledge-aware multispace embedding learning (KMEL) for personalized recommendation. We extend the user–item interaction graph with a specific semantic relation among items in a knowledge graph, resulting a semantic-specific user–item–item hybrid graph. With multiple semantics in the knowledge graph, the proposed KMEL derives multiple semantic spaces. In each semantic space, items are connected with the corresponding semantic relation; therefore, the connections vary in different semantic spaces. Due to the independence of semantics, it is intuitive to explore the embedding compensation among the semantic spaces, including the interaction space. KMEL attempts to model users’ interests across semantic structures to leverage the valuable knowledge information. It extracts the positive impact of semantics from multiple independent semantic spaces. Specifically, the high-order semantic correlation between items is built respectively in multiple independent semantic spaces, aggregated by propagating semantic correlation to embed users’ interests in each semantic space. Then, users’ interests in multiple semantic domains are adaptively integrated as a whole to recommend items. The contributions of this work are summarized as follows.

- We leverage diverse semantic correlations among items to compensate users’ sparse interaction records to mine users’ interests for recommendations;
- We propose a knowledge-aware multispace embedding learning model that respects users’ interests on each semantic and learns user embedding in a manner of divide-and-conquer across multiple semantic spaces;
- We demonstrate the effectiveness of the proposed KMEL model by experiments and corresponding analysis on two real-world datasets.

## 2. Related Work

The proposed KMEL model is related to collaborative filtering, graph-based recommendation, and knowledge-aware recommendation models. We review the literature and highlight the difference to the proposed KMEL model.

### 2.1. Collaborative Filtering

Collaborative filtering [12,13,14] as a core strategy has been widely applied in recommendation scenarios due to its simple and practical effectiveness. CF-based recommendation models exploit users’ interaction records to mine users’ interests and recommend items to users having similar interests to their owners, which do not require additional prior knowledge for personalized recommendation. Matrix factorization (MF) [12] pioneers a learnable CF model that performs recommendation by interacting user and item embeddings. MF is further developed by involving deep architecture in embedding learning [15,16,17,18] and interaction function [19]. Despite its effectiveness, CF models meet obstacles in capturing users’ interests since the personalized recommendation faces severe data sparsity and cold-start problems. In order to alleviate the problem, related studies [20,21,22] introduce diverse side information to enrich the clue for interest mining, such as social networks [20], knowledge graphs [2,10], and item content information [22], aiming at enhancing the semantic association when interacting between users and items. This work emphasizes the role of higher-order semantic associations between items and strives to leverage semantic knowledge to promote interest mining and personalized recommendation.

### 2.2. Graph-Based Recommendation

Subsequent studies figure out that rich collaborative signals remain in users’ interactions which naturally forms a heterogeneous graph with user and item nodes. With this hypothesis, graph-based recommendation models [15,16,17] propagate user and item characters layer-by-layer on the interaction graph and naturally integrate higher-order collaborative signals to model users’ interest. GCMC [15] models the effect of interactions on users and items using a graph convolutional layer on the interaction graph to encode their embeddings. PinSage [16] applies multiple graph convolutional layers on the graph to perform image recommendation. DGCF [18] builds the fine-grained user–item relationship concerning users’ intents to perform graph convolution for recommendation. NGCF [17] recursively performs information propagation on the user–item interaction graph to extract higher-order collaborative signals hidden in interactions for personalized recommendation. A light graph convolution [23] is proposed on interaction graph to model users’ interests by simplifying transformation, nonlinear activation in graph learning. Wu et al. [24] explored a context-aware graph convolution on the user–item interaction graph to digest the collaborative signals among users, items, and contexts into interaction estimation. These models aim to use heterogeneous collaborative signals hidden in users’ interactions as much as possible to embed users and items for personalized matching. Inspired by their success, this work strives to further investigate users’ interaction graph with an additional knowledge graph to deal with the interaction sparsity issue on capturing users’ interests.

### 2.3. Knowledge-Aware Recommendation

The rich semantics among items have been actively explored to mitigate cold-start and data sparsity issues. Knowledge graph, as a piece of standard auxiliary information, is introduced to bridge the semantic gap among interactions [10,11,25]. Ripplenet [11] propagates users’ interests along with the high-order paths on the user–item–entity graph by extending heterogeneous interaction graph with a knowledge graph to model semantic collaborative signals into embeddings of users and items. It employs user-interacted items as seed nodes to propagate users’ interests layer-by-layer on the knowledge graph to learn users’ embeddings. KGAT [10] utilizes a knowledge-aware neighbor-aggregation mechanism to encode user interests and introduces an attention mechanism to aggregate neighbors adaptively. Zhu et al. [26] built history interest and potential intent respectively from users’ clicked sequence and path connectivity in KGs to jointly embed users. It investigates signals from both item space and their connection space of KG. Since knowledge graph takes hybrid semantics, the diverse knowledge hidden in the graph is required to be addressed elaborately. High-order mining is easily hindered by complex semantics for personalized recommendation. The role of semantics rather than high-order user-item semantic associations on the knowledge graph should be taken into account. AIMN [25] performs pairwise user-item matching in multiple knowledge-aware attribute-level and merges the attribute-level interactions to the final score for personalized ranking. Huang et al. [27] explored multityped user–item interactive patterns with coupled graph learning on both social-aware user space and knowledge-aware item space. Users are built by the interacted items with the specific pattern and items are aggregated on all the related interactive patterns. Differently, this work strives to mine users’ interests from interaction space and multiple semantic spaces of independent relations in KG for personalized recommendation.

## 3. Methodology

**Figure 1:**
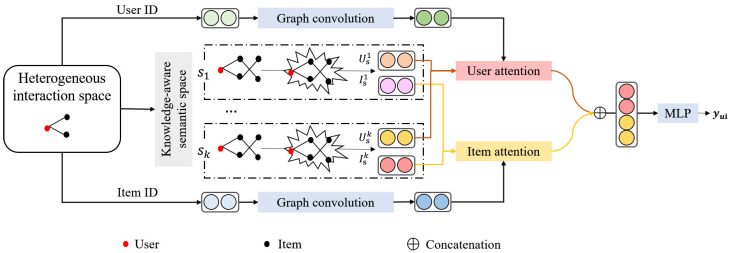
Framework of the proposed knowledge-aware multispace embedding learning (KMEL) for personalized recommendation.

Though there are redundant semantic correlations among items, involving item semantics shows promise as a way of explaining users’ interests. Therefore, we attempt to investigate semantic correlations among items to compensate interactions and model users’ interests. Mining users’ interests is implemented by embedding users relying on given historical records. As a promising auxiliary reference, an existing knowledge graph provides abundant semantic relations that relate to items’ attributes. It provides a valuable clue to reveal the interests of users who have interacted with them. In this work, we construct interest mining across semantic structures in view of multispace learning and propose knowledge-aware multispace embedding learning (KMEL) for personalized recommendation. It extracts collaborative signals over users’ high-order interaction paths in multiple independent semantic spaces and comprehensively models users and items using a siamese attention mechanism. Figure 1 shows the framework of the proposed KMEL model for personalized recommendation. Specifically, the high-order semantic correlation between items is extracted in multiple independent semantic spaces and aggregated by propagating semantic correlation to embed users’ interests by graph convolution in each semantic space. Then, users’ interests in multiple semantic domains are adaptively integrated as a whole to interact with items for recommendation. This section describes collaborative embedding learning on the user-item interaction graph, semantic embedding learning on the user-item-item graph in multiple independent semantic spaces, cross-space multisemantic fusion based on target-aware attention, and nonlinear interaction and recommendation as follows.

### 3.1. Collaborative Embedding Learning

The items that a user has interacted with provide evidence of the user’s personalized interests, while the users interacting with a specific item reflect the item’s audience character. The user-item interactions form a heterogeneous graph that contains rich collaborative signals. It is promising to model users and items by graph propagation for personalized recommendation. As illustrated in Figure 1, We capture the positive impact of high-order collaborative signals using a layer-by-layer interest propagation mechanism on the heterogeneous user–item interaction graph. Considering that users have varying interest degrees for items, it is necessary to take varying contributions of neighbors in building users and items with graph propagation. We adopt an attention-based graph convolutional layer to encode users and items by performing neighbour aggregation.

Following current mainstream recommendation strategies, we initialize user ID and item ID with high-dimensional embeddings $e_{u}^{\left(0\right)},e_{i}^{\left(0\right)}\in R_{d}$, where $R_{d}$ is a d-dimensional interest embedding space. The collaborative interest propagation from interacted item *i* to user *u* is defined as

(1) $$m_{u\leftarrow i}^{\left(1\right)}=f\left(e_{u}^{\left(0\right)},e_{i}^{\left(0\right)},\alpha_{ui}\right)$$

where $m_{u\leftarrow i}$ is the collaborative signals propagated from item *i* to user *u*, $f\left(\right)$ represents the interest propagation function, and $\alpha_{ui}$ denotes the learnable contribution parameter of item *i* to user *u*. The propagation function $f\left(\right)$ is defined as

(2) $$m_{u\leftarrow i}^{\left(1\right)}=\alpha_{ui}We_{i}^{\left(0\right)},\;\;\;\alpha_{ui}=\frac{\exp(\sigma \left(att^{T}\left[We_{u}^{\left(0\right)}⊕We_{i}^{\left(0\right)}\right]\right))}{\sum_{k\in N_{u}}\exp(\sigma \left(att^{T}\left[We_{u}^{\left(0\right)}⊕We_{k}^{\left(0\right)}\right]\right))}$$

where $\alpha_{ui}$ is learned by a single-layer attention network with parameter att. The propagation layer performs a softmax normalization to produce the neighbor contribution degree $\alpha_{ui}$ for the following embedding aggregation. *W* is a learnable linear transformation matrix, ⊕ represents concatenation operation, and σ() is the LeakyReLU activation function. The propagated collaborative signals from interacted items $i\in N_{u}$ of user *u* are aggregated to update its embedding as

(3) $$e_{u}^{\left(1\right)}=\sigma \left(m_{u\leftarrow u}^{\left(1\right)}+\sum_{i\in N_{u}}m_{u\leftarrow i}^{\left(1\right)}\right)$$

where $e_{u}^{\left(1\right)}$ represents the first-order collaborative embedding of user *u*. We stack multiple propagation layers to model higher-order collaborative embedding for user *u*. The higher-order collaborative embedding attempts to capture the effect of long-path user-item collaborative signals hidden in users’ interactions on user interest modeling. We stack *L* interest propagation layers to propagate collaborative signals for user *u* in its L-hop neighbors.

(4) $$e_{u}^{\left(l\right)}=\sigma \left(m_{u\leftarrow u}^{\left(l\right)}+\sum_{i\in N_{u}}m_{u\leftarrow i}^{\left(l\right)}\right)$$

where l=1,2,…,L indexes the embedding on layer *l*. We perform the same operation on item *i* to derive the higher-order collaborative embedding of item *i* correspondingly as

(5) $$e_{i}^{\left(l\right)}=\sigma \left(m_{i\leftarrow i}^{\left(l\right)}+\sum_{u\in N_{i}}m_{i\leftarrow u}^{\left(l\right)}\right)$$

The collaborative embeddings of users and items on the *L*th layer of the interest propagation network are represented as $U_{c}$ and $I_{c}$. The collaborative embedding reflects the metainterest of users and items, i.e., behavior of users with similar interests and audience of items with similar attributes. We utilize the collaborative embedding $U_{c}$ and $I_{c}$ to guide the subsequent modeling of semantic interest embedding of users and items.

### 3.2. Semantic Embedding Learning

This work investigates semantic correlations among items as a knowledge clue to aid users’ interactions and mine users’ interests for personalized recommendation. Considering the complex and diverse semantics in the knowledge graph, the proposed KMEL model constructs users’ interests across semantic spaces to leverage valuable knowledge information. It extracts semantic positive effects from multiple independent semantic spaces $s_{1},s_{1},\ldots ,s_{K}$. We align the interaction graph to the corresponding knowledge graph forming a hybrid graph and explore the user–item–item semantic structure in each independent semantic space to capture a semantic embedding for users and items.

In each semantic space $s_{k}$, we extract semantic collaborative signals in the hybrid graph to model users and items on the specific semantic domain. Since the items located in the same semantic space appear to be relatively compact in terms of semantics, we employ a naive graph convolution to aggregate neighbors in the hybrid graph to learn the semantic embeddings for users and items. On the hybrid graph, embeddings are also initialized with the same ID in Section 3.1, as $h_{u}^{\left(0\right)}=e_{u}^{\left(0\right)},h_{i}^{\left(0\right)}=e_{i}^{\left(0\right)}$. The semantic interest propagation is defined as

(6) $$h_{u}^{\left(l+1\right)}=\sigma \left(\sum_{i\in N(u)}\frac{1}{c_{iu}}h_{i}^{\left(l\right)}W^{\left(l\right)}+b^{\left(l\right)}\right)$$

(7) $$h_{i}^{\left(l+1\right)}=\sigma \left(\sum_{j\in N(i)}\frac{1}{c_{ji}}h_{j}^{\left(l\right)}W^{\left(l\right)}+\sum_{u\in N(i)}\frac{1}{c_{ui}}h_{u}^{\left(l\right)}W^{\left(l\right)}+b^{\left(l\right)}\right)$$

where $h_{u}^{\left(l+1\right)}$ and $h_{i}^{\left(l+1\right)}$ are the updated semantic embeddings of user *u* and item *i* at layer l+1 in semantic space *k*, respectively, N(u),N(i) represents the neighbor set of user *u* and item *i*, respectively, $b^{\left(l\right)}$ represents the bias coefficient of the *l*th layer propagation network, $c_{ji}$ is the product of the square root of node degrees $c_{ji}=\sqrt{\left|N(j)\right|}\sqrt{\left|N(i)\right|}$. On each layer, interest propagation aggregates neighbor users u∈N(i) from the interaction graph and neighbor items j∈N(i) from the knowledge graph to update embedding $h_{i}$ for item *i*. For user *u*, the propagation aggregates the neighbor items j∈N(u) the user interacts with to update the embedding $h_{u}$.

In each semantic space $s_{k}$, we capture high-order semantic collaborative signals between user-items by performing multi-layer interest propagation on the hybrid graph. We use $U_{s}^{k}$ and $I_{s}^{k}$ to represent the semantic embeddings of user *u* and item *i* on the *L*th layer in the semantic space $s_{k}$, which reveals the corresponding semantic preference of users and attribute distribution of items. Semantic embeddings from different semantic spaces reflect the diverse semantic preferences of users. With the same semantic interest propagation on the semantic-specific hybrid graph structures, the proposed KMEL derives a set of knowledge-aware semantic embeddings $U_{s}^{1}$, $U_{s}^{2}$, …, $U_{s}^{k}$ for users and $I_{s}^{1}$, $I_{s}^{2}$, …, $I_{s}^{k}$ for items.

### 3.3. Cross-Space Multisemantic Fusion

Till now, the proposed KMEL builds collaborative embeddings $U_{c}$ and $I_{c}$, and semantic embeddings $U_{s}^{1}$, $U_{s}^{2}$, …, $U_{s}^{k}$ and $I_{s}^{1}$, $I_{s}^{2}$, …, $I_{s}^{k}$. Multiple semantic embeddings capture users’/items’ preferences/attributes in different semantic spaces. As illustrated in Figure 1, we utilize the collaborative embedding $U_{c}$ and $I_{c}$ to guide the cross-space embedding fusion to learn an integrated embedding for users and items.

Considering that the various importance of these semantics for modeling users’ interests varies from user to user, we introduce a target-aware attention mechanism to learn the contribution of different semantics for modeling users’ interests, referred as importance degree.

(8) $$\alpha_{k}^{u}=softmax\left(ReLU\left(W_{att}\left(U_{c}⊕U_{s}^{k}\right)+b_{att}\right)\right)$$

(9) $$\alpha_{k}^{i}=softmax\left(ReLU\left(W_{att}\left(I_{c}⊕I_{s}^{k}\right)+b_{att}\right)\right)$$

where $\alpha_{k}^{u}$ represents the importance of user u’s semantic embedding $U_{s}^{k}$ in semantic space $s_{k}$ on modeling his/her complete interests. Similarly, $\alpha_{k}^{i}$ represents the importance of item i’s semantic embedding $I_{s}^{k}$ in semantic space $s_{k}$ to model its entire attribute. $W_{att}$ and $b_{att}$ are the learnable weights and bias coefficients of a single-layer attention network. We perform a softmax operation to normalize the importance degree of semantics. The collaborative embeddings $U_{c}$ and $I_{c}$ of users/items participate in the importance estimation of embeddings in other semantic spaces with their semantic-specific embeddings $U_{s}^{1}$, $U_{s}^{2}$, …, $U_{s}^{k}$ and $I_{s}^{1}$, $I_{s}^{2}$, …, $I_{s}^{k}$, resulting in varying importance degrees on semantics. Then, we aggregate the semantic embeddings across multiple spaces to construct an integrated embedding for users and items.

(10) $$h_{u}=\sum_{U_{s}^{k}\in \left\{U_{c},U_{s}^{1},U_{s}^{2},\ldots U_{s}^{n}\right\}}\alpha_{k}^{u}U_{s}^{k}$$

(11) $$h_{i}=\sum_{I_{s}^{k}\in \left\{I_{c},I_{s}^{1},I_{s}^{2},\ldots I_{s}^{n}\right\}}\alpha_{k}^{i}I_{s}^{k}$$

where $h_{u}$ and $h_{i}$ represent the integrated embeddings of user *u* and item *i* across multiple semantic spaces.

### 3.4. Nonlinear Interaction and Recommendation

For the pairwise matching of user *u* and item *i*, we concatenate their embeddings $h_{u}$ and $h_{i}$ as the pairwise interaction feature. The interaction feature contains matching information between user preferences and item attributes. We leverage a typical MLP network to filter further the nonlinear interaction correlation between user *u* and item *i* and predict the interest degree of user *u* on item *i*.

(12) $${\hat{y}}_{ui}=MLP\left(h_{u}⊕h_{i}\right)$$

where ${\hat{y}}_{ui}$ represents the predicted interest degree of user *u* on item *i*, MLP() is a standard nonlinear interaction function. The proposed KMEL ranks the items *i* with the highest interest degree and recommends top-K items for user *u*.

For the model optimization on user/item initialization and model parameters, we employ conventional log loss [28,29] and Adam optimizer to train the model as follows.

(13) $$L=\frac{-1}{\left|R^{+}\right|+\left|R^{-}\right|}\left[\sum_{\left(u,i\right)\in R^{+}}\log\left({\hat{y}}_{ui}\right)+\sum_{\left(u,i\right)\in R^{-}}\log\left(1-{\hat{y}}_{ui}\right)\right]+\lambda {∥\theta ∥}^{2}$$

where $R^{+}$ and $R^{-}$ represent all positive and negative samples in the training set, respectively, $|R^{+}|$ and $|R^{-}|$ are the number of positive and negative samples. During optimization, we employ $L_{2}$ regularization and dropout strategy to prevent model overfitting.

## 4. Experiments

We conduct experiments to verify the effectiveness of the proposed KMEL model for personalized recommendation. With the experiments, We aim to answer the following research questions.

- **RQ1** Compared to advanced recommendation models, how does the proposed KMEL perform?
- **RQ2** How does knowledge signals extracted from multiple semantic spaces affect the performance?
- **RQ3** How does the model hyperparameters work in KMEL?

### 4.1. Experimental Settings

#### 4.1.1. Dataset Description

Experiments are performed on Amazon-Book [30] and Yelp2018 [10] datasets for personalized recommendation, which have varying sparsity and domain knowledge. Table 1 summarizes the statistics of the experimental datasets.

- **Amazon-Book.** We selected Amazon-book from the widely used product dataset Amazon-review, which has a relatively high sparsity. We kept the users and items with at least 10 interactions to guarantee the reliability of the dataset. Considering the possible significance to a specific domain, three relations Subjects, Author, Character were selected from those given by the dataset to construct semantic spaces. With the three relations, the entity size aligned from the knowledge graph to items is large enough to mine semantic signals for modeling users’ interests.
- **Yelp2018.** Yelp2018 is a dataset sampled from the field of music applications. Similarly, we kept the users and items with at least 10 interactions for experiments. Relations Categories and Music were employed to construct semantic spaces. The relations also provided enough entity to items for modeling users’ interests.

In addition to user–item interactions, the proposed KMEL model builds multiple independent semantic spaces on the hybrid interaction–knowledge graph. The interaction–knowledge graph includes a large number of user–item–item triples, each of which is composed of an interacted user–item pair and an item–item pair taking the same semantic relation in the given knowledge graph. We built item–item connections with specific knowledge associations in the datasets. On Amazon-book and Yelp2018 datasets, two items are connected in a specific semantic space when they take the same tail entity in the knowledge graph with the semantic relation. We randomly selected 80% of the user-interacted items as positive samples in the training set, and the remaining 20% as the test set [10]. In the training set, we randomly matched a negative sample for each positive sample to participate in the model optimization [10]. In the test set, we randomly selected 100 negative samples for each positive sample of users to test the recommendation performance [28].

#### 4.1.2. Evaluation Metrics

The performances were evaluated with the commonly used Normalized Discounted Cumulative Gain at rank K (NDCG@K) and Recall@K on Top-*K* recommendation lists. We set *K* to be 10 without specification, i.e., we mainly evaluated the recommendation performance on the Top-10 items in the recommendation lists of users. For the datasets, we show the average recommendation performance based on both metrics on all the users in the test set.

#### 4.1.3. Baselines

We compare the proposed KMEL model with ID-based (NCF [28]), graph-based (NGCF [17], GCMC [15]), and knowledge-based (CKE [2], RippleNet [11], KGAT [10]) models for personalized recommendation.

- **NCF [28]** constructs a multilayer deep network to perform nonlinear user–item interactions, aiming to capture hidden nonlinear collaborative signals between users and items for recommendation. It represents a simplified KMEL model with only ID and nonlinear interaction function;
- **GCMC [15]** utilizes a graph convolutional encoder to embed users and items and feeds them into a nonlinear decoder to predict users’ interests in items. It performs the same as the collaborative embedding channel in KMEL;
- **NGCF [17]** recursively performs interest propagation on users’ interaction graph to extract higher-order collaboration signals for embedding users and items. It It additionally encodes relations to GCMC, however, only the interaction relation is investigated;
- **CKE [2]** investigates knowledge base of items to enrich latent embeddings of items and performs interaction between the enhanced item embedding with the naive latent embedding of users for pairwise matching. Knowledge enriches only item representation in CKE, but both users and items in KMEL;
- **RippleNet [11]** treats the items interacted by users as seeds and aggregates high-order semantic signals through path propagation of the seeds over a knowledge graph to learn embeddings.It involves neighbors in KG to help propagate interests while KMEL investigate both neighbors and the corresponding various relations;
- **KGAT [10]** designs knowledge-aware attention on neighbors by graph convolution to involve semantic collaborative signals of the knowledge graph into embeddings. Compared to the fine-grained attention of KGAT, the proposed KMEL applies only semantic attention to each space.

#### 4.1.4. Parameter Settings

We implemented the proposed KMEL model with the deep learning framework pytorch. The embedding size of users and items was fixed to 64, and the batch size was set to 1024. All model parameters were initialized with Gaussian distribution. We adjusted the learning rate in the range $\left[0.0001,0.001,0.01,0.1\right]$ and searched $L_{2}$ regularization strength in the range $\left[10^{-6},10^{-5},\ldots ,10^{-1},1\right]$ to prevent overfitting. The interest propagation depth of graph convolution in the proposed KMEL model were tuned in {1,2,3,4}. Without specification, all the comparison models adopted the same hyperparameter settings to compare their recommendation performance for fairness.

### 4.2. Performance Comparison (RQ1)

**Figure 2:**
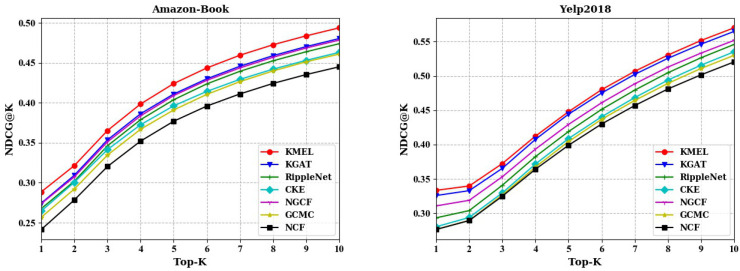
Performance comparison of Top-K recommendations by NDCG@K on Amazon-Book and Yelp2018 datasets, K=1,2,…,10.

The proposed KMEL is compared with NCF, GCMC, NGCF, CKE, RippleNet and KGAT on the Amazon-Book and Yelp2018 datasets by NDCG@10 and Recall@10. Figure 2 shows the performance comparison of the Top-K recommendation lists of the proposed KMEL and its comparisons. Table 2 provides a detailed comparison among them by NDCG@10 and Recall@10, which can be observed from the performances:

- The performance of KMEL w.r.t NDCG and Recall consistently outperformed its comparisons on the Amazon-Book and Yelp2018 datasets. By NDCG@10, the proposed KMEL achieved 2.79% and 0.94% improvement over the strongest baseline on the Amazon-Book and Yelp2018 datasets, respectively. Such performance improvement proves the effectiveness of the proposed KMEL in modeling users’ interests. The proposed KMEL is capable of finding semantic correlations among items to aid collaborative embedding learning and alleviating the data sparsity issue for personalized recommendation.
- The poor recommendation performance of NCF on the two datasets compared to other models proves the effectiveness of graph-based interest propagation and even knowledge propagation for mining users’ interests. In detail, the performance improvement of GCMC and NGCF over NCF demonstrates the significance of collaborative signals hidden in the interaction graph on revealing interests. The performances of CKE and RippleNet further illustrate the valuable role of knowledge to enrich users’ representations and comprehensively model users’ interests.
- NGCF performed better than GCMC on both datasets, demonstrating the positive role of high-order collaborative signals in modeling users’ interests. Both NGCF and KGAT explore higher-order collaborative signals to embed users, with a difference of knowledge extension in KGAT. KGAT introduced a knowledge graph to aggregate higher-order knowledge structures. Compared with NGCF, KGAT achieved better performance, which verifies the positive impact of semantics in knowledge graph to mine users’ interests.
- CKE, RippleNet, and KGAT showed an improved recommendation performance compared to NCF, GCMC, NGCF, due to the valuable knowledge structure information from the knowledge graph in building users’ interests. RippleNet outperformed CKE on both datasets, which indicates that introducing multihop neighbor items in a path-propagation manner is relatively effective for learning users’ interests, while the regularization-based method may not fully utilize the rich semantics of items. Compared with RippleNet, the performance of KGAT on both datasets shows that the embedding propagation method can utilize the rich semantics of items more effectively than the path-based and regularization-based models. Further, the attention mechanism in KGAT not only improves the interpretability of the recommendation results and further improves the recommendation performance.
- Compared with GCMC and NGCF, the excellent performance of the proposed KMEL on both datasets demonstrates the capability of semantic correlations among items to promote learning users’ interests. Meanwhile, KMEL outperformed RippleNet and KGAT, proving the effectiveness of the cross-space multisemantic structures in modeling users’ interests for personalized recommendation.

### 4.3. Knowledge-Aware Semantics (RQ2)

#### 4.3.1. Impact of Independent Semantic Structures

**Figure 3:**
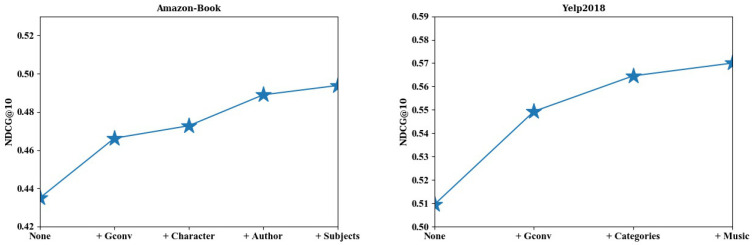
Recommendation performance of relevant submodels (+Gconv,+Character, +Author, +Subjects, +Categories, +Music) compared to that of MLP (+None) on Amazon-Book and Yelp2018 datasets by NDCG@10.

Here, we attempt to evaluate the role of multiple independent semantic structures on modeling users’ interests for recommendation. MLP, as the base model, performs interaction on IDs of users and items, which is employed subsequently to explore the increment of each independent semantic structure on interest learning. Specifically, we first conducted recommendation by MLP, i.e., the castrated KMEL, removing the graph convolution layer and all semantic structures. Then, we introduced the aforementioned collaborative embedding learning with graph convolution (+Gconv) and semantic embedding learning on semantic structures (+Character, +Author, +Subjects, +Categories, +Music), respectively. Figure 3 shows the recommendation performance of the relevant submodels (+Gconv, +Character, +Author, +Subjects, +Categories, +Music) compared to that of MLP (+None) by NDCG@10. On both datasets, it can be observed that the recommendation performance gradually improved by involving more semantics, and the proposed KMEL attained the best performance on recommendation when all the semantic structures were introduced. This demonstrates the complementary role of collaborative and knowledge embeddings on mining users’ interests. Additionally, the performance also illustrates the varying impact of semantics on recommendation. This is reasonable, since the importance of semantics varies in revealing users’ interests. Some semantics derive a relatively apparent improvement on performance, while others bring a relatively small improvement. Taking the Amazon-Book dataset as an example, the semantics of book author greatly improves the performance compared with other semantics. This coincides with the reality that readers tend to prefer books written by their favorite authors.

#### 4.3.2. Impact of Target-Aware Multispace Fusion

**Figure 4:**
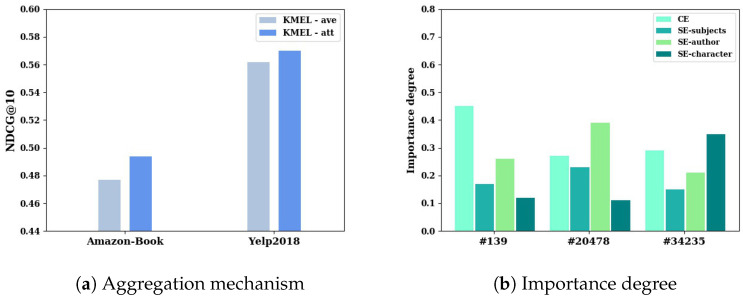
Performance comparison on aggregation by NDCG@10 with (**a**) linear (ave) and target-aware (att) aggregation mechanisms, and (**b**) importance degree of collaborative embeddings (CE) and semantic embeddings of semantics (SE-character, SE-author, SE-subjects) for users #139, #20478 and #34235 from Amazon-Book dataset.

Experiments were performed to measure the impact of the multispace fusion mechanism on mining users’ interests. We compared the recommendation performance of KMEL with linear average aggregation (ave) to that with target-aware attentive aggregation (att). Figure 4a shows the experimental performance of the proposed KMEL with average and target-aware aggregation mechanisms on Amazon-Book and Yelp2018 datasets by NDCG@10. The results show that the target-aware attention-based aggregation outperformed linear aggregation on both datasets, demonstrating the advantage of the target-aware attention mechanism to aggregate multiple spaces on embedding learning adaptively. Since users’ interests are inherently complex, global linear aggregation cannot fit users’ varying personalized interest distribution. The target-aware attention mechanism can model the importance of semantics on revealing users’ interests, which helps effectively improve the recommendation performance.

The importance degrees of semantics are evaluated by the attention mechanism. Additionally, in order to further track the effect of attention mechanism on user embedding learning, we randomly select users #139, #20478 and #34235 from Amazon-Book dataset to show the importance degree of spaces in Figure 4b. We observe the variation among these users on semantics. For user #139, collaborative embedding (CE) contributes more to modeling the user’s interests than other semantics. The other two users have relatively strong preferences on different semantics, respectively. The importance degree varies among semantics even varies among users. The varying importance degrees support the conclusion that semantics contribute different to a specific user, and the proposed KMEL is capable of building adaptive aggregation with the target-aware attention mechanism.

### 4.4. Study of KMEL (RQ3)

To measure the impact of hyperparameters on model recommendation performance, we perform experiments on interaction manner and propagation depth on Amazon-Book and Yelp2018 datasets.

#### 4.4.1. Effect of Nonlinear Interaction

**Figure 5:**
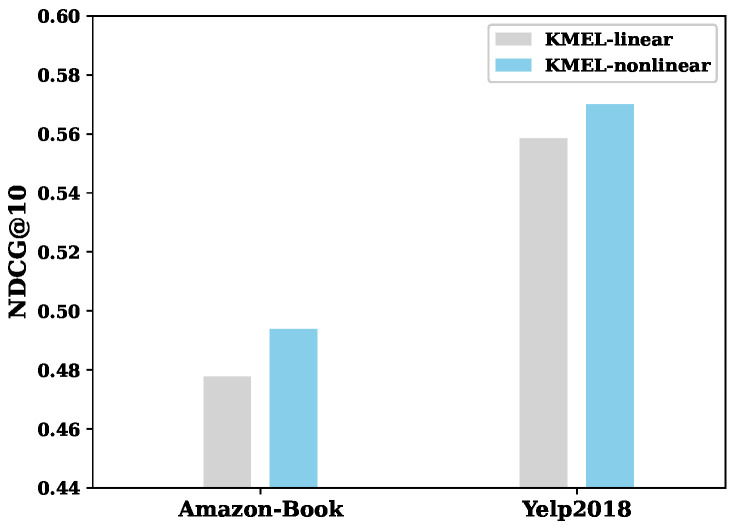
Performance of the proposed KMEL with linear and nonlinear interaction on Amazon-Book and Yelp2018 datasets.

User interests and item attributes are diverse and complex, making their interaction hard to predicte. To measure the role of interaction manner on recommendation performance, we performed KMEL with linear and nonlinear interaction functions on Amazon-Book and Yelp2018 datasets. The inner product of user–item pair conducted standard linear interaction and the MLP layer performed typical nonlinear interaction. Figure 5 shows the recommendation performance of KMEL with linear and nonlinear interaction functions by NDCG@10. It can be seen that the nonlinear interaction function performed better than the linear interaction. This proves that the nonlinear correlation inherently exists between users and items in interaction. The proposed KMEL leverages the advantage of MLP on nonlinear mapping to perform complex user–item interactions for personalized recommendation.

#### 4.4.2. Effect of Propagation Depth

We adjusted the propagation depth of graph convolution on both collaborative signals and semantic signals in the range of {1,2,3,4} to explore the effect of propagation depth of the embedding learning layer. We used KMEL-1 to characterize the model with single-layer propagation and KMEL-2,3,4 to characterize models with more depths. Table 3 summarizes the performance of the proposed KMEL with varying propagation depth by NDCG@10 and Recall@10. It shows that as the propagation depth increases, the recommendation performance gradually increases until it reaches the optimal. The optimal depths of the proposed KMEL on Amazon-Book and Yelp2018 datasets are at 3-layers for KMEL-3 and 2-layers for KMEL-2, respectively. This difference is attributed to the varying characters of the datasets, especially the difference in data sparsity. Deep propagation is required on the relatively sparse Amazon-Book dataset to capture relatively more collaborative signals on embedding users’ interests. Meanwhile, we observe that KMEL-1 outperformed other baselines in most cases, further demonstrating the positive effect of semantic knowledge on modeling users’ interests. Considering effectiveness and efficiency, the proposed KMEL adopts 2-layer propagation with graph convolution to learn embeddings for recommendation.

## 5. Conclusions

We have proposed a knowledge-aware multispace embedding learning model for personalized recommendation. The proposed KMEL extracts collaborative signals from multiple independent semantic structures and adaptively integrates collaborative and semantic signals to predict users’ interests with a target-aware aggregation. The proposed KMEL uses the semantic correlation among items to learn users’ interests, which coincides with the reality that knowledge systems exist and impact users’ interests. Extensive experiments on two real-world datasets demonstrate the effectiveness of the proposed KMEL on modeling users’ interests with multiple semantic knowledge. Personalized recommendation tasks have always faced heavy data sparsity and cold-start issues. Existing knowledge acts to explain various relations in the view of causal or codependent relationships. For example, a user likes a movie due to the famous actor. This movie–actor knowledge provides a significant clue to explain users’ interests. Valuable knowledge helps a comprehensive understanding of the world, which would be a promising way to mine and build users’ interests as a future direction.

## Figures and Tables

**Table 1:** Statistics of the datasets in experiments.

		Amazon-Book	Yelp2018
	#Users	70,679	45,919
User-Item Interactions	#Items	24,915	45,538
	#Interactions	847,733	1,185,068
	#Entities	88,572	90,961
Knowledge Graph	#Relations	39	42
	#Triplets	2,557,746	1,853,704

**Table 2:** Performance comparison by NDCG@10 and Recall@10.

	Amazon-Book		Yelp2018	
	NDCG@10	Recall@10	NDCG@10	Recall@10
NCF	0.4451	0.5105	0.5204	0.4163
GCMC	0.4609	0.5274	0.5302	0.4354
NGCF	0.4785	0.5419	0.5517	0.4401
CKE	0.4631	0.5206	0.5351	0.4408
RippleNet	0.4739	0.5391	0.5458	0.4502
KGAT	**0.4804**	**0.5493**	**0.5647**	**0.4532**
KMEL	**0.4938**	**0.5581**	**0.5709**	**0.4593**
%Improve	2.79%	1.6%	0.94%	1.36%

**Table 3:** Performance of the proposed KMEL with varying propagation depth by NDCG@10 and Recall@10.

	Amazon-Book		Yelp2018	
	NDCG@10	Recall@10	NDCG@10	Recall@10
KMEL-1	0.4751	0.5244	0.5515	0.4344
KMEL-2	0.4938	0.5581	**0.5709**	**0.4594**
KMEL-3	**0.4970**	**0.5584**	0.5701	0.4580
KMEL-4	0.4941	0.5564	0.5694	0.4583

## Data Availability

Not applicable.

## References

[1] Sun Z., Guo Q., Yang J., Fang H., Guo G., Zhang J., Burke R. (2019). Research commentary on recommendations with side information: A survey and research directions. Electron. Commer. Res. Appl..

[2] Zhang F., Yuan N.J., Lian D., Xie X., Ma W.Y. Collaborative knowledge base embedding for recommender systems. Proceedings of the 22nd ACM SIGKDD International Conference on Knowledge Discovery and Data Mining.

[3] Huang J., Zhao W.X., Dou H., Wen J.R., Chang E.Y. Improving sequential recommendation with knowledge-enhanced memory networks. Proceedings of the 41st International ACM SIGIR Conference on Research & Development in Information Retrieval.

[4] Wang H., Zhang F., Zhao M., Li W., Xie X., Guo M. Multi-task feature learning for knowledge graph enhanced recommendation. Proceedings of the World Wide Web Conference.

[5] Fan W., Ma Y., Li Q., He Y., Zhao Y.E., Tang J., Yin D. (2019). Graph Neural Networks for Social Recommendation. Proceedings of the World Wide Web Conference, WWW 2019, San Francisco, CA, USA, 13–17 May 2019.

[6] Ehrlinger L., Wöß W. Towards a definition of knowledge graphs. Proceedings of the SEMANTiCS (Posters Demos SuCCESS), Leipzig, Germany, 13–14 September 2016.

[7] Gomez-Perez J.M., Pan J.Z., Vetere G., Wu H. (2017). Enterprise knowledge graph: An introduction. Exploiting Linked Data and Knowledge Graphs in Large Organisations.

[8] Li C., Hu L., Shi C., Song G., Lu Y. (2021). Sequence-aware Heterogeneous Graph Neural Collaborative Filtering. Proceedings of the 2021 SIAM International Conference on Data Mining (SDM), Virtual Event, 29 April–1 May 2021.

[9] Hu B., Shi C., Zhao W.X., Yu P.S. (2018). Leveraging Meta-path based Context for Top- N Recommendation with A Neural Co-Attention Model. Proceedings of the 24th ACM SIGKDD International Conference on Knowledge Discovery & Data Mining, KDD 2018, London, UK, 19–23 August 2018.

[10] Wang X., He X., Cao Y., Liu M., Chua T.S. Kgat: Knowledge graph attention network for recommendation. Proceedings of the 25th ACM SIGKDD International Conference on Knowledge Discovery & Data Mining.

[11] Wang H., Zhang F., Wang J., Zhao M., Li W., Xie X., Guo M. Ripplenet: Propagating user preferences on the knowledge graph for recommender systems. Proceedings of the 27th ACM International Conference on Information and Knowledge Management.

[12] Koren Y., Bell R., Volinsky C. (2009). Matrix factorization techniques for recommender systems. Computer.

[13] Hu Y., Koren Y., Volinsky C. Collaborative filtering for implicit feedback datasets. Proceedings of the 2008 Eighth IEEE International Conference on Data Mining.

[14] Wang C., Zhu H., Zhu C., Qin C., Xiong H. Setrank: A setwise bayesian approach for collaborative ranking from implicit feedback. Proceedings of the AAAI Conference on Artificial Intelligence.

[15] Van den Berg R., Kipf T.N., Welling M. (2017). Graph Convolutional Matrix Completion. arXiv.

[16] Ying R., He R., Chen K., Eksombatchai P., Hamilton W.L., Leskovec J. Graph convolutional neural networks for web-scale recommender systems. Proceedings of the 24th ACM SIGKDD International Conference on Knowledge Discovery & Data Mining.

[17] Wang X., He X., Wang M., Feng F., Chua T.S. Neural graph collaborative filtering. Proceedings of the 42nd International ACM SIGIR Conference on Research and Development in Information Retrieval.

[18] Wang X., Jin H., Zhang A., He X., Xu T., Chua T.S. Disentangled graph collaborative filtering. Proceedings of the 43rd International ACM SIGIR Conference on Research and Development in Information Retrieval.

[19] He X., Du X., Xiang W., Feng T., Chua T.S. Outer Product-based Neural Collaborative Filtering. Proceedings of the Twenty-Seventh International Joint Conference on Artificial Intelligence IJCAI-18.

[20] Jamali M., Ester M. A matrix factorization technique with trust propagation for recommendation in social networks. Proceedings of the Fourth ACM Conference on Recommender Systems.

[21] Wang H., Zhang F., Hou M., Xie X., Guo M., Liu Q. Shine: Signed heterogeneous information network embedding for sentiment link prediction. Proceedings of the Eleventh ACM International Conference on Web Search and Data Mining.

[22] Sun Y., Yuan N.J., Xie X., McDonald K., Zhang R. (2017). Collaborative intent prediction with real-time contextual data. ACM Trans. Inf. Syst..

[23] He X., Deng K., Wang X., Li Y., Zhang Y., Wang M. Lightgcn: Simplifying and powering graph convolution network for recommendation. Proceedings of the 43rd International ACM SIGIR Conference on Research and Development in Information Retrieval.

[24] Wu J., He X., Wang X., Wang Q., Chen W., Lian J., Xie X. (2022). Graph convolution machine for context-aware recommender system. Front. Comput. Sci..

[25] Yang R., Jian M., Shi G., Wu L., Xiang Y. (2021). Attribute-Level Interest Matching Network for Personalized Recommendation. Chinese Conference on Pattern Recognition and Computer Vision (PRCV).

[26] Qiannan Z., Zhou X., Wu J., Tan J., Guo L. A Knowledge-Aware Attentional Reasoning Network for Recommendation. Proceedings of the AAAI Conference on Artificial Intelligence.

[27] Huang C., Xu H., Xu Y., Dai P., Xiao L., Lu M., Bo L., Xing H., Lai X., Ye Y. Knowledge-aware coupled graph neural network for social recommendation. Proceedings of the 35th AAAI Conference on Artificial Intelligence (AAAI).

[28] He X., Liao L., Zhang H., Nie L., Hu X., Chua T.S. Neural collaborative filtering. Proceedings of the 26th International Conference on World Wide Web.

[29] Xue F., He X., Wang X., Xu J., Liu K., Hong R. (2019). Deep item-based collaborative filtering for top-n recommendation. ACM Trans. Inf. Syst..

[30] He R., McAuley J.J. (2016). Ups and Downs: Modeling the Visual Evolution of Fashion Trends with One-Class Collaborative Filtering. Proceedings of the 25th International Conference on World Wide Web, WWW 2016, Montreal, QC, Canada, 11–15 April 2016.

